# Low Water Activity Induces the Production of Bioactive Metabolites in Halophilic and Halotolerant Fungi

**DOI:** 10.3390/md9010043

**Published:** 2010-12-27

**Authors:** Kristina Sepcic, Polona Zalar, Nina Gunde-Cimerman

**Affiliations:** Department of Biology, Biotechnical Faculty, University of Ljubljana, Vecna pot 111, SI-1000 Ljubljana, Slovenia; E-Mails: kristina.sepcic@bf.uni-lj.si (K.S.); polona.zalar@bf.uni-lj.si (P.Z.)

**Keywords:** hypersaline environments, black yeast, NaCl, secondary metabolites, hemolysis, antibacterial activity

## Abstract

The aim of the present study was to investigate indigenous fungal communities isolated from extreme environments (hypersaline waters of solar salterns and subglacial ice), for the production of metabolic compounds with selected biological activities: hemolysis, antibacterial, and acetylcholinesterase inhibition. In their natural habitats, the selected fungi are exposed to environmental extremes, and therefore the production of bioactive metabolites was tested under both standard growth conditions for mesophilic microorganisms, and at high NaCl and sugar concentrations and low growth temperatures. The results indicate that selected halotolerant and halophilic species synthesize specific bioactive metabolites under conditions that represent stress for non-adapted species. Furthermore, adaptation at the level of the chemical nature of the solute lowering the water activity of the medium was observed. Increased salt concentrations resulted in higher hemolytic activity, particularly within species dominating the salterns. The appearance of antibacterial potential under stress conditions was seen in the similar pattern of fungal species as for hemolysis. The active extracts exclusively affected the growth of the Gram-positive bacterium tested, *Bacillus subtilis*. None of the extracts tested showed inhibition of acetylcholinesterase activity.

## 1. Introduction

As crystalline salt (NaCl) is generally considered to be hostile to most forms of life, it has been used for centuries as a food preservative. However, halophilic and halotolerant microorganisms can contaminate food that is preserved with salt, and they can also inhabit natural hypersaline environments around the world, such as salt lakes and solar salterns. These microorganisms can adapt to extreme concentrations of NaCl, and often to high concentrations of other ions [1], and also to extremely low water activity (*a_w_*), due to the chemical bonding of water to NaCl [2]. The great majority of studies of halophilic and halotolerant microorganisms have been dedicated to halophilic bacteria and archaea [3–5], and to only one eukaryotic species, the alga *Dunaliella salina* [6], as it has been considered that other eukaryotic organisms cannot adapt to these extreme conditions [7].

Black yeasts were first reported to be active inhabitants of brine in solar salterns in 2000 [1]. Since then, many new fungal species and species previously known only as contaminants of food preserved with high concentrations of salt or sugar have been discovered in hypersaline environments around the globe [8–12]. These new ecological findings have not only improved our understanding of complex microbial processes in these natural hypersaline environments, but they have also contributed to the not yet fully acknowledged demonstration that food can be contaminated with potentially mycotoxigenic fungi via the salt (or sugar) used as the preservative.

Surprisingly, many fungal species that were first found in hypersaline environments were later detected in polythermal Arctic glaciers [8,13,14]. Thus, despite the extreme differences in physical conditions between glaciers and solar salterns exposed to the heat and UV of strong sunlight, the common critical parameter for species found both in Arctic glaciers and in Mediterranean salterns is the low *a_w_*, which is associated with glaciers due to ice formation.

In general, marine organisms represent a wide source of yet undiscovered compounds that, besides unprecedented chemical structures, often possess interesting biological activities. These could find their use in medicine, pharmaceutics, industry, and other fields of human activities. The production of biologically active compounds in marine-, soil-, and food-derived fungi has been the subject of several investigations over the last 10 years (see also below); however, there have been no such investigations focusing on halophilic and halotolerant fungi. Thus, the aim of the present study was to investigate stable fungal communities both in hypersaline waters of solar salterns and in subglacial ice in relation to the synthesis of bioactive metabolites with hemolytic, antibacterial, and acetylcholinesterase (AChE) inhibition activity. The discovery of new antibiotics is one of the most important goals in biomedical research, since the appearance of multiresistant bacterial strains urged the search of new strategies for treatment of bacterial infections. As to AChE inhibitors, these substances could find their use in treating disorders like Alzheimer disease, myasthenia gravis and glaucoma. Substances exerting above-selected biological activities may also have important ecological roles, as they could serve in territorial competition, or in protection against other hypersaline species. In this regard, hemolytic and antimicrobial compounds could act against protozoa and bacteria, while AChE inhibitors might be directed against higher organisms with a developed nervous system.

As the fungi tested in this work are exposed to extremes of *a_w_* and temperature in their natural habitats, production of bioactive metabolites was tested not only under the standard growth conditions for mesophilic microorganisms (*a_w_* = 1.0, room temperature), but also at high NaCl and sugar concentrations and low growth temperatures. These ecologically relevant conditions have the potential to influence the production of known compounds or the synthesis of new, as-yet-unknown, biologically active secondary metabolites.

## 2. Results and Discussion

Until a decade ago, it was a general belief in microbial ecology that fungi cannot inhabit natural hypersaline environments. Few fungi contaminating food or other substrates characterized by low *a_w_* were recognized as “domestic extremophiles” with a general xerophilic phenotype that was determined by the water potential of the medium, rather than by the chemical nature of the solute [15]. These fungi were considered xerophilic if they grew well at an *a_w_* of ≤0.85, corresponding to 17% NaCl or 50% glucose in their growth medium. Nowadays we know that extremotolerant fungal species are present pan-globally [1,16] in hypersaline environments and also in extremely cold environments, such as Arctic glaciers and Antarctic rocks, as well as in the deep-sea. Most fungi inhabiting these extreme environments can be considered extremotolerant. Fungi from hypersaline environments do not require salt for viability, but can tolerate salt to very high concentrations (from 0 to 30% NaCl) [17]. Only few fungi display halophilic behaviour [9], since they require at least 5–10% NaCl. This trend is evident also for most fungi that inhabit cold environments, which show not just halotolerance, but also psychrotolerance, which is characterized by a wide range of cardinal temperatures [18].

Most fungi representing the core mycobiota in salterns and Arctic glaciers were previously known as food-borne species or species with no recognized primary natural niche; alternatively, they were not known to science, and consequently have been described as new [8–12,19]. Although, at present, there are a total of 140 orders of fungi known [20], tolerance for low *a_w_* is apparent in only 10, which are not near phylogenetic neighbors. In any of these particular orders, growth at decreased *a_w_* is in most cases limited to a few species, or to a single genus of an order [9], indicating a polyphyletic origin of extremotolerance in fungi, which would also imply different mechanisms of adaptation. For the present study, we selected phylogenetically different halophilic and halotolerant species (see Table 1). Amongst the selected fungi, there were the halophilic black yeast species *Hortaea werneckii*, *Phaetotheca triangularis*, and *Trimmatostroma salinum*, which represent the dominant fungal inhabitants of solar salterns worldwide [21]. As well as in salterns, the halotolerant *Aureobasidium pullulans* can be found in extremely cold environments. Recently four varieties of *A. pullulans* were described [13] that, as well as having differences in their morphology and molecular markers, also show different preferences for low *a_w_* environments. Representatives of two of these varieties were studied, *A. pullulans* var. *pullulans*, which can be found in the phyllosphere, salterns and other osmotic environments, and *A. pullulans* var. *melanogenum*, isolated from water environments and deep-sea [13]. Selected different species of the related genus *Cladosporium* are also amongst the dominant inhabitants of solar salterns [8] and they are also very common in extremely cold (Arctic) environments [18]. Seven of these species are new to science and were described only recently. The inclusion of *Wallemia* spp. in the present study is of particular importance, as until recently this genus contained only one food-borne species, *Wallemia sebi*. Recent taxonomic analyses resolved *Wallemia* into three species: *W. sebi*, *W. muriae* and *W. ichthyophaga* [9], the last of which is presently the most halophilic eukaryote known to date [22]. Both *W. ichthyophaga* and the extremely halotolerant black yeast *H. werneckii* are model organisms for studies of halotolerance in eukaryotes [23]. Several *Alternaria* and *Fusarium* species were included in the present study as well, as they probably represent new species and representatives of melanized fungi, which have been isolated from both glaciers and salterns [15]. Although the production of biologically active compounds in fungi has been well documented, particularly due to their biotechnological importance [24–26] and mycotoxigenic potential [27], to our knowledge, extremophilic fungi have been completely overlooked in such studies. It is of note that extracts and metabolites from fungi belonging to different genera included in this study have already been tested for their biological activities. These fungi belong to species inhabiting mesophilic terrestrial environments, which show their best production of bioactive metabolites under mesophilic conditions. For example, species of the genus *Fusarium* have been reported to have antifungal [28–31], antibacterial [32–34], cytotoxic [29,32,35–38], and anti-inflammatory [36] activities. Metabolites from *Cladosporium* spp. have also been shown to have antibacterial [39,40], antifungal, and cytotoxic [40] components, while those from *W. sebi* can cause death of brine shrimps, protozoans and cell lines [41], and show significant inhibition of bacterial growth [42]. Finally, *A. pullulans* was reported to have anticoagulant, antithrombotic, and antiviral potential [43].

### Biological Activities of the Tested Fungal Strains

Our data suggest that the hemolytic activities of the fungal strains tested are most probably associated with organic molecules. Water extracts (both crude and thermally treated) were never hemolytic, regardless of the growth conditions, suggesting that these organisms do not synthesize proteins or other polar molecules that can destroy erythrocyte membranes.

The hemolytic potential of the organic extracts (Table 2) was considerably higher if the fungi tested were exposed to conditions which would be stressful for non adapted organisms during their growth. Only 20% of the tested acetone extracts in three biological tests (9 out of 44) exerted stronger hemolysis under control conditions, and this trend was not observed in any of the methanolic extracts tested. On the contrary, increases in hemolytic activity were detected in 43% of the acetone extracts, and up to 72.7% of the methanolic extracts of fungi grown under these tested conditions. Increased salt concentrations resulted in significant increases in the hemolytic activities of both the acetone and methanolic extracts of the halophilic black yeasts *H. werneckii* and *T. salinum*, and the polar, non-melanized yeast *Rhodosporidium diovobatum*, as well as in one of the three strains of *Aureobasidium* (EXF-922). Surprisingly, this trend was even more pronounced when particularly halotolerant fungi were exposed to low temperatures or increased glucose concentrations during growth. Under these conditions, besides the above-mentioned strains, increased hemolytic potential was also detected in all of the tested strains of *Alternaria tenuissima* and *Candida parapsilosis*, in the majority of *Cladosporium* spp. and *Fusarium* spp., in all of the tested *Wallemia* and *Cryptococcus* species, and in *Pichia guilliermondii*, *Alternaria arborescens* and *Rhodotorula mucilaginosa*.

As disruption of the cell membrane is one of the mechanisms that can lead to cell death, hemolysis might be associated with some cytotoxic compounds that have already been isolated from the fungal species tested. Marine-, soil- and plant-derived *Fusarium* species, for example, contain a plethora of small cytotoxic organic molecules, like the polyketide fusarielins [29], sesterterpene neomagnicols [32] and magnicols [36], and the cyclic pentadepsipeptides sansalvamide [35] and *N*-methylsansalvamide [37]. Cytotoxicity has also been described for marine-derived *Cladosporium* sp., and was attributed to macrolide sporiolides [40], as well as in food-derived *Wallemia* spp. strains, which contain some cytotoxic compounds: *cis*-fused iso-caryopyllenes walleminol and walleminone [41], and azasteroides UCA 1064-B and UCA 1064-A [42]. Interestingly, the production of walleminol was seen to be enhanced when *W. sebi* was cultivated in media with a high sucrose concentration (20%), which is in agreement with the increased activity observed in our study under all of the non-control conditions. All of the above-mentioned compounds were active against different normal and transformed cell lines in the range of several hundreds of nanograms, to several tens of micrograms per mililiter.

As in the case of hemolysis, only organic fungal extracts, and hence no water extracts, showed antibacterial activities. These antibacterial activities of the organic extracts were enhanced when the fungi were cultivated under the non-control conditions (Table 3). However, enhancement of this kind of biological activity was not as pronounced as for hemolysis, and was seen for 34% of acetone, and only 22% of methanolic extracts. In comparison, 27% of the tested extracts inhibited bacterial growth more readily under control conditions. The appearance of antibacterial potential under non-control conditions was seen in the similar spectrum of fungal species as for hemolysis, and comprised *A. pullulans* (var. *melanogenum* and *Aureobasidium* sp.), selected *Cladosporium* (*C. halotolerans*, *C. psychrotolerans* and *C. oxysporum*), *Alternaria*, *Fusarium* and all the tested *Cryptococcus* and *Wallemia* strains, *H. werneckii*, *P. triangularis*, *T. salinum*, *C. parapsilosis* and *P. guilleiermondii*. All of the active extracts exclusively affected the growth of the Gram-positive bacterium *B. subtilis*, but were practically ineffective against the Gram-negative bacterium *E. coli* (not shown). Inhibitory activities of controls (pure solvents) were found to be zero.

Antibacterial activity has already been reported for some of the fungal species tested, and as for hemolysis, this was attributed to small organic metabolites excreted by these fungi. These comprise a sesterterpene, neomagnicol B [32] and a cyclic tetrapeptide, JM47 [33] from marine-derived *Fusarium* sp., 5-hydroxymethyl 2-furanocarboxylic acids (Sumiki’s acids) [39] and marcolides sporiolides A and B [40] from marine-derived strains of *Cladosporium*, and azasteroides UCA 1064-B and UCA 1064-A from *W. sebi* [42]. A phenolic natural product known as hortein, which is the only compound that has been isolated from *H. werneckii* to date, did not show any inhibitory activity against *B. sublitis*, *E. coli* and other bacterial and fungal strains tested [44].

None of the tested extracts showed inhibition of the activity of AChE, an enzyme responsible for the cleavage of the neurotransmitter acetylcholine in the synaptic cleft, suggesting that neurotoxicity does not have a role in the defense strategies of the fungal strains tested.

**Figure 1:**
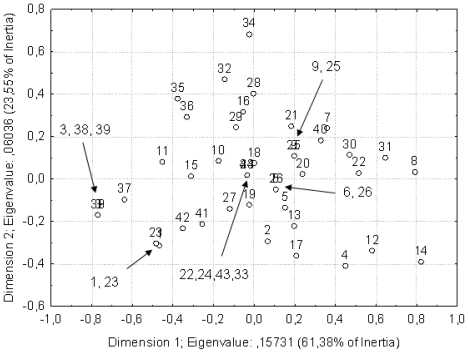
The data on hemolytic and antibacterial activity of extract analyzed with correspondence analysis. Strains are numbered as in Table 1, first column.

The data on hemolytic and antibacterial activity of the extracts were analyzed by correspondence analysis to see whether any grouping was possible. The first two principal components described approximately 70% of the variation of the data (Figure 1). According to the performed analysis, the central part of the core group is represented by extremotolerant species, which are subgrouped into three clusters. One of them consists of dominant halotolerant fungi inhabiting salterns, the other one contains dominant psychrotolerant species from the glaciers, and the third one halophilic species, which are more specialized and less widely represented in the hypersaline environments. Overlap between them represents generalist, extremotolerant and ubiquitious species. Outside the core group are fungi differing in ecology and distribution: On one hand, *Saccharomyces cerevisiae*, used as the control, and on the other hand, species known for their limited distribution and stenotolerance in relation to temperature or osmotic stress.

## 3. Experimental Section

### 3.1. Fungal Sources

The hypersaline waters used for the isolation of selected halophilic and halotolerant fungi originated from solar salterns in Slovenia (Adriatic Sea) [1], Israel (Red Sea), Spain, France (Mediterranean Sea), Portugal, Namibia, Dominican Republic, Puerto Rico (Atlantic Ocean), and from the Dead Sea, the Great Salt Lake (Utah, USA), and the Enriquillo Salt Lake (Dominican Republic) [21]. All of the Arctic fungi were isolated from glaciers in the Kongsfjorden, Svalbard and Spitzbergen archipelagoes in Norway [14].

The selected strains and experimental growth conditions for these fungi are given in Table 1. All of the fungi are deposited in the Culture Collection of Extremophilic Fungi (EXF) of the Department of Biology, Biotechnical Faculty, University of Ljubljana, Slovenia. Out of 44 tested strains, 43 were halophilic and halotolerant, and one strain was used as a control (*Saccharomyces cerevisiae*).

### 3.2. Cultivation of Fungi

All but two of the fungal strains were cultivated on Yeast Nitrogen Base (YNB) agar medium without (*a_w_* = 0.993) and with the addition of 10% NaCl (YNB-NaCl, *a_w_* = 0.930) or 40% glucose (YNB-S, *a_w_* = 0.928) (see Table 1). For the low NaCl concentration, two species of the genus *Wallemia* (*Wallemia muriae* and *Wallemia ichthyophaga*) were cultivated at 5% NaCl (*a_w_* = 0.962) and 10% NaCl, respectively, due to their obligate need for NaCl in the medium; NaCl was then added to 17% (*a_w_* = 0.873), with glucose tested for these two species at 40% and 55%, respectively. YNB, YNB-NaCl and YNB-S plates were incubated for up to 14 weeks at 30 °C, and YNB plates additionally at temperatures lowered to 4 °C or 10 °C, according to tests of the growth of these fungi under these conditions. All of the fungi except the yeasts were grown on the top of sterile cellophane layered on the listed agar media.

### 3.3. Preparation of Extracts

After incubation, the biomass of the various fungi was scraped from the surface of 5–10 agar plates and freeze dried. This material was divided into three parts, with each homogenized by grinding and extracted overnight by continuous shaking with deionized water (4 °C), methanol or acetone (37 °C) (material:solvent, 1:2, w:v). The aqueous extracts were then centrifuged (30 min; 12,092× *g*; 4 °C), and the supernatants were divided into two, with one aliquot stored at −20 °C until further use. The other aliquot was heated for 15 min at 100 °C to denature proteins and other thermolabile compounds, and centrifuged as before, with the supernatant stored at −20 °C until further use. The extractions with the organic solvents were filtered, and the filtrates were dried using a rotary vacuum pump. The dry material obtained was dissolved in 1–2 mL of 96% ethanol. The concentrations of all of the extracts was determined gravimetrically, and adjusted to 5 mg dry weight/mL by addition of the appropriate solvent (water or ethanol, respectively) prior to the biological assays.

### 3.4. Estimation of Antimicrobial Activity

Antimicrobial activity was tested against two bacterial strains obtained from the Culture Collection of Extremophilic Microorganisms at the Department of Biology, Biotechnical Faculty, University of Ljubljana, Slovenia: a Gram positive *Bacillus subtilis* (EXB-V68), and a Gram negative *Escherichia coli* (EXB-V1). Antimicrobial activities were evaluated using the standard agar-diffusion test. Briefly, these bacteria were allowed to grow overnight in Luria Bertani (LB) medium, and their concentrations were determined by turbidimetric assay. After that, an appropriate volume of bacterial culture was added to LB nutrient agar previously cooled to 42 °C. The final concentrations of these bacteria were approximately 5 × 10^5^ CFU/mL. Twenty milliliters of inoculated medium were poured into Petri dishes and incubated at 4 °C for 24 h. The circles of agar (Φ = 1 cm) were cut out from the cooled medium. To estimate the antibacterial activity, 100 μL of each extract were pipetted into the holes cut in the inoculated medium, with the samples incubated at 37 °C for 24 h. The diameters of the inhibition zone were then measured. As controls, the inhibitory activities of the pure solvents (water and ethanol) were checked as well. Every measurement was repeated at least in three parallel samples.

### 3.5. Estimation of Hemolytic Activity

Hemolytic activity was measured by a turbidimetric method [45]. Twenty microliters of each extract or of the pure solvents (water and ethanol) were added to 180 μL bovine erythrocyte suspension with an apparent absorbance of 0.5 at 650 nm. The final concentration of the fungal extract in the test was 0.5 mg/mL. The decrease in the apparent absorbance due to hemolysis was recorded over 20 min, at 650 nm, using a Kinetic Microplate Reader (Dynex Technologies, U.S.). This was used to determine the time necessary for 50% hemolysis: *t*_50_. All of the experiments were performed at 25 °C, with every measurement repeated at least three times.

### 3.6. Inhibition of Acetylcholinesterase

AChE activity was measured according to the Ellman method [46], using acetylthiocholine iodide (1 mM) as the substrate in 100 mM potassium phosphate buffer, pH 7.4, at 25 °C, and electric eel AChE as the source of enzyme (6.25 U/mL, Sigma). Hydrolysis of acetylthiocholine iodide was followed on a Kinetic Microplate Reader (Dynex Technologies, USA) at 412 nm. AChE inhibition was monitored for 5 min for each extract; and the latter were assayed in the final concentration of 100 μg/mL. The effects of the pure solvents (water and ethanol) on the assay were also monitored. The ethanol that was used for the preparation of organic extracts did not exceed 5% of the total volume of the reaction mixtures, with all of the readings corrected for their appropriate blanks, and every measurement repeated at least three times.

### 3.7. Multivariate Statistical Analyses

The qualitative data on hemolytic and antibacterial activity of fungal extract were analyzed with correspondence analysis using the Statistical programs (StatSoft, Inc., Tulsa, Oklahoma, USA).

## 4. Conclusions

In conclusion, it appears that selected halotolerant and halophilic species synthesize specific bioactive metabolites under conditions that represent stress for non-adapted species. Furthermore, specialization of adaptation at the level of the chemical nature of the solute that lowers the *a_w_* of the medium can be observed, since increased salt concentrations resulted in distinct increases in the hemolytic activity, particularly with the dominant ecological group from the salterns, the halophilic black yeast *H. werneckii*, *T. salinum*, the Arctic-inhabiting *Aureobasidium* sp. and *A. pullulans* var. *melanogenum* and filamentous *Wallemia* spp. Other species tested showed more general xerophilic behavior, which was characterized by increased synthesis of bioactive compounds when they were exposed to increased glucose concentrations or low temperatures, both of which induced a lowering of the *a_w_*. It is tempting to speculate that besides having a protective role or helping in territorial competition, secondary bioactive metabolites might have roles in yet-to-be described adaptation mechanisms of halophilic and halotolerant fungi under conditions of lowered *a_w_*. Isolation and structure elucidation of those metabolites is in progress and will be published elsewhere.

## Figures and Tables

**Table 1:** Strains, their origins, and growth conditions for metabolite production.

Strain no. *	Species	Strain accession no.	Origin	Temperature (°C)		Glucose (w/v%)		NaCl (w/v%)	
				↓Temp	↑Temp	↓Glc	↑Glc	↓NaCl	↑NaCl
1	*Acremonium strictum*	EXF-2277	Hypersaline water from salterns; Sečovlje, SI	4	30	2	30	0	10
2	*Alternaria arborescens*	EXF-2340	Soil from salterns covered with gypsum; Sečovlje, SI	4	30	2	40	0	10
3	*Alternaria infectoria*	EXF-2332	Hypersaline water from salterns; Sečovlje, SI	10	30	2	30	0	10
4	*Alternaria tenuissima*	EXF-2318	Hypersaline water from salterns; Sečovlje, SI	4	30	2	40	0	10
5	*Alternaria tenuissima*	EXF-2329	Hypersaline water from salterns; Sečovlje, SI	4	30	2	40	0	10
6	*Alternaria tenuissima*	EXF-2338	Hypersaline water from salterns; Sečovlje, SI	4	30	2	40	0	10
7	*Aureobasidium pullulans* var. *pullulans*	EXF-150	Hypersaline water from salterns; Sečovlje, SI	4	30	2	40	0	10
8	*Aureobasidium pullulans* var. *melanogenum*	EXF-3382	Deep-sea (4500 m depth); Sea of Japan, JP	4	30	2	40	0	10
9	*Aureobasidium* sp.	EXF-922	Subglacial ice from sea water; Svalbard, NO	4	30	2	40	0	10
10	*Candida parapsilosis*	EXF-517	Hypersaline water from salterns; Sečovlje, SI	10	30	2	40	0	0
11	*Candida parapsilosis*	EXF-1574	Glacial ice; Svalbard, NO	10	30	2	40	0	10
12	*Cladosporium cladosporioides*	EXF-381	Hypersaline water from salterns; Sečovlje, SI	10	30	2	40	0	10
13	*Cladosporium dominicanum*	EXF-732 **^T^**	Hypersaline water; lake Enriquilo, DO	10	30	2	40	0	10
14	*Cladosporium fusiforme*	EXF-449 **^T^**	Hypersaline water from salterns; Sečovlje, SI	4	30	2	40	0	10
15	*Cladosporium halotolerans*	EXF-572 **^T^**	Hypersaline water from salterns; salterns; NA	10	30	2	40	0	10
16	*Cladosporium halotolerans*	EXF-2513	Glacial ice, Svalbard, NO	10	30	2	40	0	10
17	*Cladosporium langeronii*	CBS 189.54 **^NT^**	Man, mycosis; BR	10	30	2	40	0	10
18	*Cladosporium oxysporum*	EXF-2246	Ice, Staubaier glacier, AT	4	30	2	40	0	10
19	*Cladosporium psychrotolerans*	EXF-391 **^T^**	Hypersaline water from salterns; Sečovlje, SI Slovenia	4	30	2	40	0	10
20	*Cladosporium salinae*	EXF-335 **^T^**	Hypersaline water from salterns; Sečovlje, SI	10	30	2	40	0	10
21	*Cladosporium sphaerospermum*	EXF-385	Hypersaline water from salterns; Sečovlje, SI	10	30	2	40	0	10
22	*Cladosporium spinulosum*	EXF-334 **^T^**	Hypersaline water from salterns; Sečovlje, SI	10	30	2	40	0	10
23	*Cladosporium velox*	EXF-466	Hypersaline water from salterns; Sečovlje, SI	4	30	2	40	0	10
24	*Cryptococcus albidus*	EXF-3363	Glacial ice; Svalbard, NO	4	30	2	40	0	10
25	*Cryptococcus liquefaciens*	EXF-3409	Glacial ice; Svalbard, NO	4	30	2	40	0	10
26	*Cryptococcus magnus*	EXF-3360	Glacial ice; Svalbard, NO	4	30	2	30	0	10
27	*Fusarium* aff. *equiseti*	EXF-2275	Hypersaline water from salterns; Sečovlje, SI	10	30	2	40	0	10
28	*Fusarium graminearum*	EXF-2254	Hypersaline water from salterns; Sečovlje, SI	4	30	2	40	0	10
29	*Fusarium verticilloides*	EXF-2276	Hypersaline water from salterns; Sečovlje, SI	10	30	2	40	0	10
30	*Hortaea werneckii*	EXF-225	Hypersaline water from salterns; Sečovlje, SI	10	30	2	40	0	10
31	*Phaeotheca triangularis*	EXF-206	Hypersaline water from salterns; Sečovlje, SI	4	30	2	40	0	10
32	*Pichia guilliermondii*	EXF-518	Hypersaline water from salterns; Sečovlje, SI	4	30	2	40	0	10
33	*Pichia guilliermondii*	EXF-1496	Seawater; Svalbard, NO	4	30	2	40	0	10
34	*Rhodosporidium babjevae*	EXF-513	Hypersaline water from salterns; Sečovlje, SI	4	30	2	40	0	10
35	*Rhodosporidium diobovatum*	EXF-3361	Glacial ice; Svalbard, NO	4	30	2	40	0	10
36	*Rhodotorula mucilaginosa*	EXF-1630	Glacial ice; Svalbard, NO	4	30	2	40	0	10
37	*Saccharomyces cerevisiae*	EXF-531	Unknown	10	30	2	40	0	
38	*Trichosporon mucoides*	EXF-1444	Hypersaline water from salterns; Eilat, IL	10	30	2	30	0	10
39	*Trichosporon mucoides*	EXF-3366	Glacial ice; Svalbard, NO	10	30	2	40	0	10
40	*Trimmatostroma salinum*	EXF-295 **^T^**	Hypersaline water from salterns; Sečovlje, SI	10	30	2	40	0	10
41	*Wallemia ichthyophaga*	EXF-994 **^NT^**	Hypersaline water; Sečovlje salterns, SI	10	30	55	55	10	17
42	*Wallemia muriae*	EXF-951 **^NT^**	Hypersaline water; Sečovlje salterns, SI	10	30	40	40	5	17
43	*Wallemia sebi*	CBS 818.96 **^NT^** (EXF-958)	Sunflower seed; SE	22	30	2	40	0	10

EXF, Culture Collection of Extremophilic Fungi, Biotechnical Faculty, Department of Biology, Večna pot 111, Ljubljana, Slovenia; T, ex-type strain; NT, ex-neotype strain; CBS, Centraalbureau voor Schimmelcultures, Fungal Biodiversity Center, Utrecht, The Netherlands.

* Strains are numbered in succession; numbers in the first column correspond to the strain numbers shown in the correspondence analysis in Figure 1.

**Table 2:** Hemolytic activity of extracts of selected halophilic and halotolerant fungi.

Species	Strain	Acetone extracts				Methanolic extracts			
		Control	10% NaCl	Low T	40% glucose	Control	10% NaCl	Low T	40% glucose
*Acremonium strictum*	EXF-2277	−		−	−	−	−	−	−
*Alternaria arborescens*	EXF-2340	−	−	−	−	−	−	−	+
*Alternaria infectoria*	EXF-2332	−	−	−	−	−	−	−	−
*Alternaria tenuissima*	EXF-2318	−	−	−	+++	−	−	++	+
*Alternaria tenuissima*	EXF-2329	++	−	−	−	−	−	+++	++
*Alternaria tenuissima*	EXF-2338	−	−	+++	−	−	−	+++	++
*Aureobasidium pullulans* var. *pullulans*	EXF-150	+++	−	−	++	++	−	+++	++
*Aureobasidium pullulans* var. *melanogenum*	EXF-3382	+++	+++	−	−	++	−	++	++
*Aureobasidium* sp.	EXF-922	−	+++	−	+	−	+++	+++	++
*Candida parapsilosis*	EXF-517	−	−	+++	++	−	−	+++	−
*Candida parapsilosis*	EXF-1574	−	−	+++	−	−	−	+++	+
*Cladosporium cladosporioides*	EXF-381	+++	−	−	−	++	−	+	++
*Cladosporium dominicanum*	EXF-732	−	−	+	−	++	−	++	++
*Cladosporium fusiforme*	EXF-449	+++	−	−	+	++	−	+++	−
*Cladosporium halotolerans*	EXF-572	−	−	+++	−	+	−	+++	−
*Cladosporium halotolerans*	EXF-2513	++	ND		−	ND	ND	+++	++
*Cladosporium langeronii*	CBS 189.54	+	−	−	−	−	−	++	++
*Cladosporium oxysporum*	EXF-2246	−	−	−	++	−	−	+++	++
*Cladosporium psychrotolerans*	EXF-391	+	−	−	−	−	−	+++	+++
*Cladosporium salinae*	EXF-335	+++	−	−	−	++	−	+++	++
*Cladosporium sphaerospermum*	EXF-385	+++	−	+++	−	++	−	+++	++
*Cladosporium spinulosum*	EXF-334	++	−	−	−	+	−	−	++
*Cladosporium velox*	EXF-466	−	−	−	−	−	−	−	−
*Cryptococcus albidus*	EXF-3363	−	−	+	−	−	+++	+++	++
*Cryptococcus liquefaciens*	EXF-3409	−	−	+++	−	−	−	+++	+++
*Cryptococcus magnus*	EXF-3360	++	−	−	−	+++	−	+++	−
*Fusarium* aff*. equiseti*	EXF-2275	−	−	−	−	−	−	+++	++
*Fusarium graminearum*	EXF-2254	−	−	+++	+++	−	−	+++	+++
*Fusarium verticilloides*	EXF-2276	−	−	−	−	−	−	+++	+++
*Hortea werneckii*	EXF-225	−	+++	−	−	−	++	+++	+++
*Phaeotheca triangularis*	EXF-206	+++	+++	−	−	++	++	++	−
*Pichia guilliermondii*	EXF-518	+++	+++	+++	−	+	−	+++	−
*Pichia guilliermondii*	EXF-1496	−	−	+++	−	−	−	−	+++
*Rhodosporidium babjevae*	EXF-513	+++	+++	+++	+++	++	−	++	++
*Rhodosporidium diobovatum*	EXF-3361	−	+++	+++	−	−	++	+++	−
*Rhodotorula mucilaginosa*	EXF-1630	−	−	++	++	−	−	+++	+++
*Saccharomyces cerevisiae*	EXF-531	−	−	−	−	−	−	++	−
*Trichosporon mucoides*	EXF-1444	−	−	−	−	−	−	−	−
*Trichosporon mucoides*	EXF-3366	−	−	−	−	−	−	−	−
*Trimmatostroma salinum*	EXF-295	−	+++	−	−	−	+++	+++	++
*Wallemia ichthyophaga*	EXF-994	−	−	−	+++	−	−	−	+
*Wallemia muriae*	EXF-951	−	−	−	−	−	−	++	−
*Wallemia sebi*	EXF-958	−	−	+++	−	−	−	+++	−

Degree of hemolysis evaluated according to the half-time of hemolysis (*t*_50_) (time in which 50% of erythrocytes were lysed): +++, *t*_50_ 0–5 min; ++, *t*_50_ 5–10 min; +, *t*_50_ 10–15 min; −, t_50_ > 20 min;

Light gray shading, hemolytic activity increased upon growth of fungi under non-control conditions;

Final concentrations of extracts in all the tests were 0.5 mg/mL;

Water extracts were all negative, and are not included in the table;

ND, not determined; Low T, low temperature.

**Table 3:** Antibacterial activity of extracts of selected halophilic and halotolerant fungi on *B. subtilis.*

Species	Strain	Acetone extracts				Methanolic extracts			

		Control	10% NaCl	Low T	40% glucose	Control	10% NaCl	Low T	40% glucose
*Acremonium strictum*	EXF-2277	−	−	−	+	−	−	+	−
*Alternaria aborescens*	EXF-2340	++	++	−	−	+	−	+	+
*Alternaria infectoria*	EXF-2332	−	−	−	−	−	−	−	−
*Alternaria tenuissima*	EXF-2329	+	+	−	−	+	+	−	−
*Alternaria tenuissima*	EXF-2318	+	+	−	+	+	+	−	+
*Alternaria tenuissima*	EXF-2338	−	+	−	−	+	+	−	+
*Aureobasidium pullulans* var. *pullulans*	EXF-150	++	+	−	−	+	+	−	−
*Aureobasidium pullulans* var. *melanogenum*	EXF-3382	+	+	++	−	+	+	+	+
*Aureobasidium* sp.	EXF-922	−	+	−	−	−	+	−	+
*Candida parapsilosis*	EXF-517	−	+	−	+	−	−	−	−
*Candida parapsilosis*	EXF-1574	−	−	−	−	−	−	−	−
*Cladosporium cladosporioides*	EXF-381	+	+	−	+	+	+	−	+
*Cladosporium dominicanum*	EXF-732	+	−	−	−	+	−	−	+
*Cladosporium fusiforme*	EXF-449	+	+	+	+	+	+	+	+
*Cladosporium halotolerans*	EXF-572	−	−	−	−	−	−	−	+
*Cladosporium halotolerans*	EXF-2513	−	−	−	−	−	−	−	+
*Cladosporium langeronii*	CBS 189.54	+	+	−	−	+	−	−	+
*Cladosporium oxysporum*	EXF-2246	++	−	−	−	−	+	−	+
*Cladosporium psychrotolerans*	EXF-391	−	+	+	−	+	−	−	−
*Cladosporium salinae*	EXF-335	+	+	+	−	+	−	−	−
*Cladosporium sphaerospermum*	EXF-385	+	+	−	−	+	−	−	−
*Cladosporium spinulosum*	EXF-334	+	++	+++	+	−	+	+++	+
*Cladosporium velox*	EXF-466	−	−	−	−	−	−	+	+
*Cryptococcus albidus*	EXF-3363	−	−	−	−	−	+	+	−
*Cryptococcus liquefaciens*	EXF-3409	+	++	+	−	−	+	+	−
*Cryptococcus magnus*	EXF-3360	+	−	+	−	+	−	−	+
*Fusarium* aff. *equiseti*	EXF-2275	+	−	−	−	+	−	+	−
*Fusarium graminearum*	EXF-2254	−	−	++	+	−	−	+	−
*Fusarium verticilloides*	EXF-2276	++	−	−	+	+	−	+++	−
*Hortea werneckii*	EXF-225	+	++	−	−	+	+	+	+
*Phaeotheca triangularis*	EXF-206	+	++	−	−	+	+	+	+
*Pichia guilliermondii*	EXF-518	++	−	−	−	−	−	−	−
*Pichia guilliermondii*	EXF-1496	+	++	−	−	+	+	−	−
*Rhodosporidium babjevae*	EXF-513	−	−	−	−	−	−	−	−
*Rhodosporidium diobovatum*	EXF-3361	−	−	−	−	−	−	−	−
*Rhodotorula mucilaginosa*	EXF-1630	−	−	−	−	−	−	−	−
*Saccharomyces cerevisiae*	EXF-531	−	−	−	−	−	−	−	−
*Trichosporon mucoides*	EXF-1444	−	−	−	−	−	−	−	−
*Trichosporon mucoides*	EXF-3366	−	−	−	−	−	−	−	−
*Trimmatostroma salinum*	EXF-295	++	+	−	+	−	+	+	−
*Wallemia ichthyophaga*	EXF-994	−	+	−	+	−	−	−	−
*Wallemia muriae*	EXF-951	−	−	+	+	−	−	−	−
*Wallemia sebi*	EXF-958	+	++	−	−	+	−	+	−

Inhibition zones (mm) as follows: +++, 11–15 mm; ++, 6–10 mm; +, 1–5 mm; −, no inhibition;

Light gray shading, antibacterial activity increased upon cultivation of fungi under non-control conditions;

Extract tested, 0.5 mg of each;

Water extracts were all negative, and are not included in the table;

Low T, low temperature.
